# Renal Impairment, *C. difficile* Recurrence, and the Differential Effect of Bezlotoxumab: A Post Hoc Analysis of Pooled Data From 2 Randomized Clinical Trials

**DOI:** 10.1093/ofid/ofaa248

**Published:** 2020-06-26

**Authors:** Yoav Golan, Herbert L DuPont, Fernando Aldomiro, Erin H Jensen, Mary E Hanson, Mary Beth Dorr

**Affiliations:** 1 Tufts Medical Center, Boston, Massachusetts, USA; 2 University of Texas School of Public Health, Houston, Texas, USA; 3 Baylor St. Luke’s Medical Center, Houston, Texas USA; 4 Hospital Fernando Fonseca, EPE – Amadora/Sintra at Portugal, Area Metropolitana de Lisboa, Carnaxide, Portugal; 5 Merck & Co., Inc., Kenilworth, New Jersey, USA

**Keywords:** bezlotoxumab, *Clostridioides* difficile, recurrence, renal dysfunction

## Abstract

**Background:**

Renal impairment is not a consistently cited risk factor for recurrent *Clostridioides difficile* infection (rCDI). We examined the association between renal impairment and rCDI and the effect of bezlotoxumab, an anti–toxin B monoclonal antibody, in reducing rCDI in participants with renal impairment.

**Methods:**

We pooled data from 2 randomized, double-blind, placebo-controlled, multicenter, phase 3 clinical trials conducted in participants receiving bezlotoxumab or placebo infusion during oral antibacterial drug treatment for CDI. We assessed the association between renal impairment and rCDI in placebo-treated participants and evaluated the effect of bezlotoxumab vs placebo in reducing rCDI among participants with renal impairment, defined as an estimated glomerular filtration rate <90 mL/min.

**Results:**

The proportion of placebo-treated participants experiencing rCDI within 12 weeks was higher in those with renal impairment (n = 919) vs those without renal impairment (n = 612; 36.6% and 27.7%, respectively; difference, 8.9%; 95% CI, 1.3% to 16.3%). Renal impairment was significantly associated with a higher rate of recurrence in placebo-treated participants lacking commonly recognized risk factors for rCDI (renal impairment as only risk factor, 28.8%; vs normal renal function and no risk factors, 12.5%; difference, 16.3%; 95% CI, 3.4% to 28.8%). Among all participants with renal impairment, the rate of rCDI was 19.5% among bezlotoxumab-treated vs 36.6% among placebo-treated participants (difference, –17.1%; 95% CI, –23.4% to –10.6%).

**Conclusions:**

This post hoc analysis adds to the literature suggesting an association of renal impairment as an independent risk factor for rCDI and provides preliminary evidence that patients with renal impairment who suffer with CDI may benefit from adjunctive treatment with bezlotoxumab.

*Clostridioides difficile* infection (CDI) is the most frequent cause of nosocomial diarrhea in adults in the United States and is increasingly associated with community-acquired gastrointestinal infections [1, 2]. Despite effective treatment of CDI with antibiotics, about 15% of patients treated for primary CDI with fidaxomicin and ~29% of patients treated with vancomycin will experience a recurrence (rCDI) [3, 4], and ~40% of patients who have a first recurrence will experience 2 or more rCDIs [5]. Much of the current CDI intervention development is focused on reducing rCDI. Given that novel interventions tend to be more costly to acquire, the ability to stratify patients by their risk of rCDI would allow the identification of patients who will greatly benefit from such intervention and in whom the cost of such interventions can be easily justified.

Widely accepted risk factors associated with rCDI include advanced age [6, 7] and history of CDI. Additional factors include concomitant systemic antibiotic exposure [8], inadequate immune response [9], and prolonged hospitalization [10]. Chronic kidney disease (CKD) was found to be associated with an increased risk of CDI and CDI-associated morbidity and mortality [11], and 3 recent studies reported that CKD may be associated with higher rates of rCDI [12–14]. As patients with renal impairment tend to be older and frequently exposed to health care, it remains unclear whether renal impairment is an independent risk factor for rCDI, particularly in those lacking other risk factors. Despite demonstrated increased morbidity and mortality from CDI among patients with renal impairment, these patients have not specifically been targeted for interventions that reduce recurrence.

Bezlotoxumab is a fully human monoclonal antibody against *C. difficile* toxin B. In phase 3 trials (MODIFY I and MODIFY II), a single infusion of bezlotoxumab was associated with a significantly lower rate of rCDI compared with placebo in adults receiving antibiotic treatment for CDI [15, 16]. The MODIFY protocols prespecified subgroup analyses of participants with risk factors associated with rCDI: age ≥65 years, prior CDI, compromised immunity, severe CDI, and infection due to a known hypervirulent strain (ribotypes 027, 078, or 244). In participants with at least 1 of these 5 risk factors, bezlotoxumab reduced the proportion of participants with rCDI compared with placebo by ~16% (relative reduction, ~43%), and the relative reduction was 54% in participants with 3 or more of these risk factors [17]. The objectives of the current analysis were to examine the association of renal impairment with rCDI and to evaluate the efficacy of bezlotoxumab in these hard-to-treat patients.

## METHODS

### Study Design and Participants

MODIFY I (NCT01241552) and MODIFY II (NCT01513239) were randomized, double-blind, placebo-controlled, multicenter, phase 3 trials that were conducted from November 2011 through May 2015 at 322 sites in 30 countries. The institutional review board or independent ethics committee at each study center approved the protocols and all amendments, and each study was conducted in accordance with Good Clinical Practice Guidelines and the Declaration of Helsinki. Written informed consent was obtained before study procedures were performed.

Adults with confirmed CDI were eligible for enrollment, including those with multiple previous episodes of CDI. Eligibility criteria were previously described [15]. Participants were receiving or planned to initiate oral antibacterial treatment (metronidazole, vancomycin, or fidaxomicin, chosen by the treating physician) prescribed for 10–14 days. CDI was diagnosed based on diarrhea (≥3 unformed bowel movements in 24 hours) and a positive stool test for toxigenic *C. difficile* or its toxins. The protocols had broad inclusion and limited exclusion criteria, permitting a comprehensive evaluation of participants with diverse underlying comorbidities and a wide range of clinical characteristics associated with a high risk for additional CDI episodes, with infections caused by >130 different strains of *C. difficile*.

### Randomization and Masking

Participants were randomized to receive a single infusion of bezlotoxumab (10 mg/kg of body weight), actoxumab (10 mg/kg; MODIFY I only), bezlotoxumab + actoxumab (10 mg/kg each), or placebo (0.9% saline). In the current analysis, we only included participants randomized to the bezlotoxumab or placebo groups. Randomization was stratified by antibacterial treatment and hospitalization status (inpatient or outpatient) at the time of randomization. The participants, investigators, study center personnel except for the pharmacist preparing the infusion, and sponsor were blinded to the randomization assignments until the trial was completed and the database was locked. Due to slight differences in appearance between bezlotoxumab and placebo, infusion bags were covered in an opaque sleeve to ensure that other study personnel and all participants remained blinded to study treatment assignment.

### Procedures

Loose stool counts (types 5, 6, and/or 7 on the Bristol Stool Chart) were recorded by participants daily through 12 weeks. New episodes of diarrhea were monitored via scheduled phone contacts between visits. If there was a new episode of diarrhea, a stool sample was collected and tested for toxigenic *C. difficile*, regardless of suspected diagnosis. Safety assessments included monitoring for infusion reactions for 24 hours after infusion, pre- and postinfusion electrocardiogram, vital sign measurements, recording of adverse events, and safety laboratory values during infusion and through week 4. Serious adverse experiences (SAEs including death) were assessed through week 12.

### End Points and Subgroup Definitions

Initial clinical cure was defined as no diarrhea for 2 consecutive days after completion of antibacterial therapy. Response was defaulted to clinical failure if therapy extended beyond 16 calendar days. Initial clinical cure was assessed in the modified intent-to-treat (mITT) population (defined as all randomly assigned participants who received study infusion, had a positive toxigenic *C. difficile* stool test, and initiated antibacterial therapy before or within 1 day after the infusion). rCDI was defined as the development of a new episode of diarrhea associated with a positive stool test for toxigenic *C. difficile* within 12 weeks following study medication infusion and was assessed in mITT participants who achieved initial clinical cure (Clinical Cure population). Additional end points included 30-day all-cause hospital readmissions (defined as a hospital readmission within 30 days of discharge); 30-day CDI-associated readmission (defined as a readmission that satisfied ≥1 of the following criteria: occurrence within 5 days after onset of a new episode of CDI; onset of a new CDI episode during the readmission; or discharge diagnosis included terms synonymous with CDI, rCDI, or pseudomembranous colitis, as recorded on the trial case report form); and proportion of participants who died within 90 days after randomization for any reason.

For this analysis, the estimated glomerular filtration rate (eGFR) was calculated using the Modified Diet in Renal Disease (MDRD) equation using the serum creatinine value at the time of randomization [18]. Each participant was categorized by renal function status at time of randomization using a binary variable based on the eGFR value: without renal impairment eGFR ≥90 mL/min/1.73 m^2^, with renal impairment eGFR <90 mL/min/1.73 m^2^. Subgroups by degree of renal function were not presented for this analysis (ie, mild, moderate, severe, and end-stage renal disease) because the number of participants in the severe and end-stage renal disease categories was small, and the power to detect a difference between treatment groups was low.

### Statistical Methods

The analysis was conducted with the pooled data set from MODIFY I and MODIFY II. As this was a post hoc analysis, there was no preplanned determination of optimal sample size. We performed 2 separate analyses. The first analysis evaluated the association between renal impairment and rCDI overall. To avoid a possible confounding by treatment, we only included placebo-treated participants in this analysis. The second analysis evaluated the efficacy of bezlotoxumab in the renal impairment group. In this analysis, we included all participants with any degree of kidney impairment.

Baseline demographic and clinical characteristics of the mITT population were summarized descriptively using frequencies and percentages. In the placebo-treated participants, observed clinical cure and rCDI rates along with unadjusted rate differences for the “with renal impairment” and “without renal impairment” subgroups were calculated. The 95% confidence intervals were based on Miettinen and Nurminen’s method without stratification [19]. Further analyses of the placebo-treated participants were conducted to understand the impact of renal impairment on the rate of rCDI when combined with additional risk factors. A logistic regression analysis was performed using a model that included terms for renal impairment in addition to the known risk factors of age ≥65 years and ≥1 prior episode of CDI within 6 months. Odds ratios and 95% CIs were calculated.

 In the subgroup of participants with renal impairment, observed clinical cure and rCDI rates, unadjusted rate differences, and 95% CIs for the rate differences comparing the placebo and bezlotoxumab treatment groups were also analyzed. The nonparametric Kaplan-Meier method was used to estimate the distribution of time to recurrence of baseline CDI episode for each treatment in the subgroup of participants with renal impairment.

## RESULTS

The mITT population included 1554 participants: 781 in the bezlotoxumab group and 773 in the placebo group. For this analysis, 1531 participants were included (919 [60.0%] in the renal impairment group and 612 [40.0%] in the no renal impairment group); 23 of the full mITT population were excluded because of missing baseline eGFR values. In the group of participants with renal impairment, 447 received bezlotoxumab and 472 received placebo. Among the 919 participants with renal impairment, 489 had mild renal impairment (53.2%, eGFR 60 to <90 mL/min 1.73 m^2^), 290 had moderate renal impairment (31.6%, eGFR 30 to <60 mL/min 1.73 m^2^), 71 had severe renal impairment (7.7%, eGFR 15 to <30 mL/min 1.73 m^2^), and 69 had end-stage renal disease (7.5%, eGFR <15 mL/min 1.73 m^2^). There were 381 (85%) and 400 (85%) participants with renal impairment who remained in the study through week 12 in the bezlotoxumab and placebo groups, respectively. A similar proportion of participants without renal impairment completed the study in both treatment groups (280 [87%] bezlotoxumab, 239 [82%] placebo). The primary reason for premature discontinuation in both treatment groups was death (n = 65 in the renal impairment group [bezlotoxumab: n = 34 of 447; placebo: n = 31 of 472] and n = 39 in the no renal impairment group [bezlotoxumab: n = 16 of 322; placebo: n = 23 of 290]).

### Baseline Characteristics and rCDI Incidence in Placebo Recipients With and Without Renal Impairment

Table 1 summarizes the baseline demographics and clinical characteristics of the placebo-treated participants by renal impairment vs no renal impairment. There was a higher percentage of participants with renal impairment who were ≥65 years of age, had a Charlson index ≥3, and had at least 1 of the 5 prespecified risk factors for rCDI compared with participants without renal impairment (Table 1).

**Table 1.** Baseline Demographics and Clinical Characteristics in the Placebo-Treated Participants (mITT Population)

	Renal Impairment Status	
Characteristics	With Impairment (n = 472), No. (%)	Without Impairment (n = 290), No. (%)
Host characteristics		
Inpatient	306 (64.8)	206 (71.0)
Outpatient	166 (35.2)	84 (29.0)
Female	278 (58.9)	164 (56.6)
≥65 y of age^a^	276 (58.5)	124 (42.8)
Immunocompromised^a^	90 (19.1)	61 (21.0)
Hepatic impairment	25 (5.3)	19 (6.6)
Charlson Index ≥3	208 (44.1)	91 (31.4)
At least 1 of 5 prespecified risk factors	369 (78.2)	205 (70.7)
*C. difficile* characteristics		
≥1 CDI episode in past 6 mo^a^	138 (29.2)	78 (26.9)
≥2 previous CDI episodes ever	85 (18.0)	38 (13.1)
Severe CDI (Zar score ≥2)^a^	72 (15.3)	52 (17.9)
Participant with a positive culture	298 (63.1)	182 (62.8)
027, 078, or 244 strain	74 (24.8)	41 (22.5)
027 strain^a^	63 (21.1)	37 (20.3)
Treatment for CDI		
Metronidazole	225 (47.7)	146 (50.3)
Vancomycin	229 (48.5)	136 (46.9)
Fidaxomicin	18 (3.8)	8 (2.8)
Use of concomitant antibiotics		
Antibiotic use during SOC	159 (33.7)	110 (37.9)
Antibiotic use after SOC	129 (27.3)	91 (31.4)
Renal impairment		
Mild (eGFR 60 to <90 mL/min 1.73 m^2^)	258 (54.6)	—
Moderate (eGFR 30 to <60 mL/min 1.73 m^2^)	149 (31.6)	—
Severe (eGFR 15 to <30 mL/min 1.73 m^2^)	31 (6.6)	—
ESRD (eGFR <15 mL/min 1.73 m^2^)	34 (7.2)	—

Abbreviations: CDI, *Clostridioides difficile* infection; eGFR, estimated glomerular filtration rate; ESRD, end-stage renal dysfunction; mITT, modified intent-to-treat; mo, months; SOC, standard of care; y, years.

^a^Predefined risk factor for recurrent CDI.

**Figure 1.**
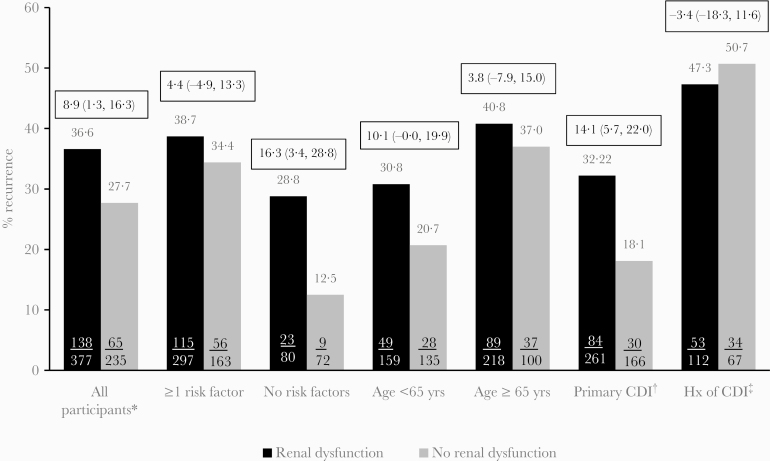
Proportion of placebo-treated participants with rCDI in all participants subset by renal impairment status and in subgroups of participants with prespecified risk factors (Clinical Cure Population). Boxes above bars are differences and 95% confidence intervals. *Clinical Cure Population; †No history of CDI within previous 6 months; ‡History of CDI within previous 6 months; risk factors for rCDI: ≥65 years of age, immunocompromised, history of CDI in previous 6 months, severe CDI (Zar score ≥2), 027 strain.

Among placebo-treated participants, there was no difference in the proportion of participants who had an initial clinical cure in the renal impairment group (79.9%) compared with those without renal impairment (81.0%; difference, –1.2%; 95% CI, –6.8% to 4.8%). In contrast, the proportion of placebo participants who experienced rCDI during the 12-week follow-up period was higher in those with renal impairment than in those without renal impairment (36.6% and 27.7%, respectively; difference, 8.9%; 95% CI, 1.3% to 16.3%) (Figure 1). When further assessing the risk of recurrence in participants based on risk factors for rCDI, renal impairment was associated with a higher rate of recurrence even in participants lacking more commonly recognized risk factors for rCDI (renal impairment as the only risk factor, 28.8%; vs normal renal function and no risk factors, 12.5%; difference, 16.3%; 95% CI, 3.4% to 28.8%). This trend was also seen in younger participants (age <65 years; 30.8% and 20.7%, respectively; difference, 10.1%; 95% CI, 0.0% to 19.9%) and in participants with primary CDI (32.2% and 18.1%, respectively; difference, 14.1%; 95% CI, 5.7% to 22.0%) (Figure 1). In elderly participants and in patients with a history of CDI, the rate of rCDI was high, and renal impairment did not appear to further increase the risk of rCDI.

Independent risk factors that were found to be associated with rCDI in univariate analyses were also included in a multivariate logistic regression. These included age ≥65 years, history of CDI in the past 6 months, and renal impairment. When modeling the odds of increased rCDI rates using logistic regression, there was significant interaction between history of CDI and renal impairment. Among participants with no prior episodes of CDI, the independent odds of recurrence for those with renal impairment were 2.0 (95% CI, 1.2 to 3.2). However, among those with a history of CDI, the odds of rCDI for those with renal impairment were 0.8 (0.4 to 1.5). Both age and CDI history remained significant in the multivariate model as well.

In a preliminary analysis, we estimated the rate of rCDI in the mild renal impairment group (eGFR 60 to <90 mL/min 1.73 m^2^) because there was a sizeable number of placebo-treated participants (n = 214); the rCDI rate was 40.2% in participants with mild renal impairment, compared with 27.7% in the no renal impairment group.

### Outcomes in Participants With Renal Dysfunction

Demographic and clinical characteristics of the study participants with renal dysfunction were similar between the bezlotoxumab and placebo groups (Table 2), and there was no difference between treatment groups in the proportion of participants who achieved initial clinical cure (Table 3). Bezlotoxumab treatment was associated with a reduction in the incidence of rCDI compared with placebo (difference, –17.1%; 95% CI, –23.4% to –10.6%) (Table 3). The results were similar in the subgroups of participants with renal dysfunction and at least 1 additional risk factor (difference, –18.5%; 95% CI, –25.7% to –11.2%), ≥1 CDI episode in the previous 6 months (difference, –18.5%; 95% CI, –30.8% to –5.5%), and age ≥65 years (difference, –20.4%; 95% CI, –28.7% to –11.8%) (Table 3). Hospital readmissions within 30 days of discharge for any reason were similar in the placebo-treated participants compared with bezlotoxumab-treated participants (27.5% and 24.5%, respectively) (Table 3). In contrast, the observed rate of CDI-associated hospital readmissions within 30 days of discharge was higher in the placebo-treated participants (13.7%) compared with bezlotoxumab-treated participants (5.0%) (Table 3). The proportions of participants who died within 90 days of randomization were similar between the 2 treatment groups (7.2% and 7.9%, respectively) (Table 3).

**Table 2.** Baseline Demographics and Clinical Characteristics by Treatment Allocation in Participants With Renal Dysfunction

Characteristics	Bezlotoxumab (n = 447), No. (%)	Placebo (n = 472), No. (%)
Host characteristics		
Inpatient	298 (66.7)	306 (64.8)
Outpatient	149 (33.3)	166 (35.2)
Female	248 (55.5)	278 (58.9)
≥65 y of age^a^	268 (60.0)	276 (58.5)
Immunocompromised^a^	94 (21.0)	90 (19.1)
Hepatic impairment	25 (5.6)	25 (5.3)
Charlson Index ≥3	201 (45.0)	208 (44.1)
At least 1 of 5 risk factors	356 (79.6)	369 (78.2)
*C. difficile* characteristics		
≥1 CDI episode in past 6 mo^a^	133 (29.8)	138 (29.2)
≥2 previous CDI episodes ever	67 (15.0)	85 (18.0)
Severe CDI (Zar score ≥2)^a^	76 (17.0)	72 (15.3)
Participant with a positive culture	282 (63.1)	298 (63.1)
027, 078, or 244 strain	55 (19.5)	74 (24.8)
027 strain^a^	48 (17.0)	63 (21.1)
Treatment for CDI		
Metronidazole	209 (46.8)	225 (47.7)
Vancomycin	222 (49.7)	229 (48.5)
Fidaxomicin	16 (3.6)	18 (3.8)
Use of concomitant antibiotics		
Antibiotic use during SOC	143 (32.0)	159 (33.7)
Antibiotic use after SOC	148 (33.1)	129 (27.3)
Renal impairment		
Mild (eGFR 60 to <90 mL/min 1.73 m^2^)	231 (51.7)	258 (54.6)
Moderate (eGFR 30 to <60 mL/min 1.73 m^2^)	141 (31.5)	149 (31.6)
Severe (eGFR 15 to <30 mL/min 1.73 m^2^)	40 (9.0)	31 (6.6)
ESRD (eGFR <15 mL/min 1.73 m^2^)	35 (7.8)	34 (7.2)

Abbreviations: CDI, *Clostridioides difficile* infection; eGFR, estimated glomerular filtration rate; ESRD, end-stage renal dysfunction; mo, months; SOC, standard of care; y, years.

^a^Predefined risk factor for recurrent CDI.

**Table 3.** CDI Outcomes by Treatment Allocation in Bezlotoxumab and Placebo Participants With Renal Impairment

Outcome	Bezlotoxumab, n/N (%)	Placebo, n/N (%)	Difference (95% CI), %
Initial clinical cure^a^	353/447 (79.0)	377/472 (79.9)	–0.9 (–6.2 to 4.3)
CDI recurrence^b^			
All patients with renal dysfunction	69/353 (19.5)	138/377 (36.6)	–17.1 (–23.4 to –10.6)
Renal dysfunction + ≥1 additional RF	57/282 (20.2)	115/297 (38.7)	–18.5 (–25.7 to –11.2)
Renal dysfunction + ≥1 CDI episode in previous 6 mo	30/104 (28.8)	53/112 (47.3)	–18.5 (–30.8 to –5.5)
Renal dysfunction + age ≥65 y	44/215 (20.5)	89/218 (40.8)	–20.4 (–28.7 to –11.8)
30-d readmissions			
All-cause	73/298 (24.5)	18/306 (27.5)	ND
Associated with CDI^c^	15/298 (5.0)	34/306 (13.7)	ND
90-d all-cause mortality^d^	36/454 (7.9)	34/475 (7.2)	ND

Abbreviations; CDI, *Clostridioides difficile* infection; d, day; mITT, modified intent-to-treat; ND, analysis not done; mo, months; RF, risk factor for rCDI (risk factors include CDI history in the past 6 months, severe CDI at baseline [per Zar score], age ≥65 years, hypervirulent strain [027, 078, or 244 ribotypes] at baseline, and being immunocompromised).

^a^mITT population.

^b^Clinical cure population.

^c^In-patient population.

^d^All patients as treated population.

**Figure 2.**
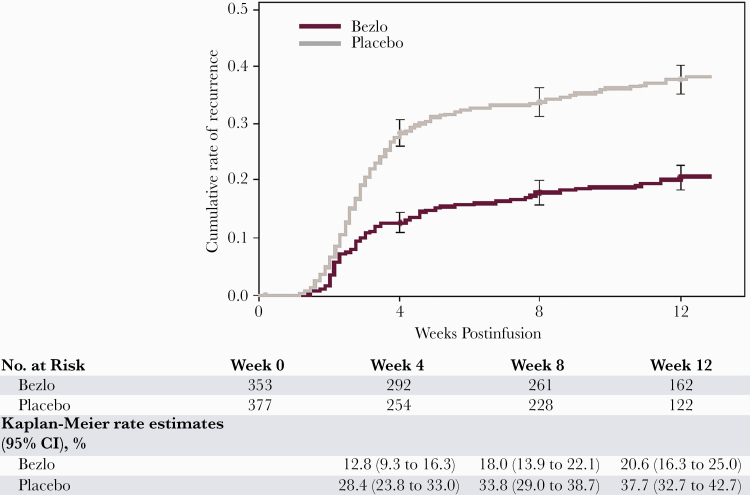
Time to *Clostridioides difficile* infection recurrence by treatment allocation in participants with renal dysfunction.

In participants with renal impairment, the majority of all recurrences (64% in the bezlotoxumab group and 76% in the placebo group) occurred within the first 4 weeks following the infusion (Figure 2). When comparing between treatment groups, the week 4, week 8, and week 12 Kaplan-Meier rCDI event rates were lower in the bezlotoxumab group than in the placebo group. Differences in the distribution of time to rCDI between the bezlotoxumab and placebo treatment groups were apparent as early as 3 weeks postinfusion and continued throughout the 12-week follow-up period.

## DISCUSSION

Previous studies have found an association between the presence of chronic kidney disease and end-stage renal disease (ESRD) and an increased risk of rCDI, higher mortality, and poor treatment responses [6, 12, 13, 20–22]. Abdelfatah et al. assessed the relationship of comorbidities with rCDI rates and showed that low eGFR (particularly in patients with stage 5 chronic kidney disease on dialysis) was significantly associated with rCDI [21]. Two separate systematic review and meta-analyses, as well as a post hoc analysis, that included participants with CKD and/or renal dysfunction demonstrated significantly increased risks of rCDI in patients with chronic kidney disease [12, 13, 20]. Although 1 study showed no association between chronic kidney disease and primary CDI, mortality and rCDI were significantly higher (by ~3 times) in patients with both CDI and chronic renal failure compared with CDI alone [22]. Despite these prior publications, an independent effect of chronic kidney disease on rCDI has not been clearly demonstrated; therefore, patients with chronic kidney disease have not been targeted for interventions to reduce future risk of CDI.

Bezlotoxumab is indicated for the prevention of rCDI in patients being treated with antibiotics for CDI who are at high risk of rCDI. Therefore, it is important to identify patients with clinically important risk factors for rCDI and to ascertain whether bezlotoxumab can provide a meaningful benefit in patients with 1 or more of these risk factors. Our post hoc analysis of placebo-treated participants enrolled in the MODIFY trials showed that renal impairment was associated with a substantial increased risk of rCDI. This was particularly evident when no other known risk factors were present. In fact, we found that even a mild degree of impairment increases a patient’s risk for rCDI. These results confirm previous reports that found an association of renal impairment with a higher rate of rCDI compared with participants with normal renal function [12–14]. Furthermore, we found that in participants without 1 of 2 major risk factors for rCDI (ie, participants aged <65 and patients with primary disease) renal dysfunction appears to be an independent risk factor for rCDI. These results support inclusion of renal impairment in the list of host characteristics that predict increased risk for rCDI.

Among these at-risk patients with renal impairment who were enrolled in the MODIFY trials, bezlotoxumab-treated participants had a significantly lower rate of rCDI compared with those who received a placebo infusion. Hence, similar to what has been demonstrated for other subgroups at high risk of rCDI (eg, the elderly, immunocompromised, patients with a history of CDI) [17], bezlotoxumab has been demonstrated to reduce future risk of CDI in a problematic patient group.

According to the Peer Kidney Care Initiative, within the first 12 months of dialysis initiation, admissions for primary CDI increased nearly 44% between 2004 and 2012 among maintenance dialysis patients, and the rates of hospitalization remain high for CDI in this population, often resulting in recurrence [23]. Because of the high risk of rCDI in these high-risk subgroups, the use of bezlotoxumab is expected to result in a reasonable number needed to treat and an improved pharmacoeconomic profile. In the current analysis, we observed a clinically important reduction in CDI-associated hospital readmissions during the 30 days postdischarge in bezlotoxumab-treated compared with placebo-treated participants with renal dysfunction.

### Limitations

This was a post hoc analysis and therefore was not powered to assess for statistical significance; the results should be interpreted in this context. Also, there could be some confounding because randomization was not stratified by degree of kidney function. Nevertheless, the data set was fairly balanced across both treatment groups, and there was a reasonable number of patients, such that the power was high. Finally, we did not separate the subgroups by degree of renal function because the numbers of participants in the severe and end-stage renal disease categories were small; additional studies are needed to assess whether the benefit of bezlotoxumab extends across the entire spectrum of renal impairment, especially those with ESRD.

## CONCLUSIONS

This post hoc analysis adds to the body of evidence suggesting an association of renal impairment as an independent risk factor for rCDI and provides preliminary evidence that patients with renal impairment suffering with CDI may benefit from adjunctive treatment with bezlotoxumab.
