# Corrigendum to: Renal Impairment, *C. difficile* Recurrence, and the Differential Effect of Bezlotoxumab: A Post Hoc Analysis of Pooled Data From 2 Randomized Clinical Trials

**DOI:** 10.1093/ofid/ofaa629

**Published:** 2021-01-20

**Authors:** Yoav Golan, Herbert L DuPont, Fernando Aldomiro, Erin H Jensen, Mary E Hanson, Mary Beth Dorr

**Affiliations:** 1 Tufts Medical Center, Boston, Massachusetts, USA; 2 University of Texas School of Public Health, Houston, Texas, USA; 3 Baylor St. Luke’s Medical Center, Houston, Texas, USA; 4 Hospital Fernando Fonseca, EPE – Amadora/Sintra at Portugal, Area Metropolitana de Lisboa, Carnaxide, Portugal; 5 Merck & Co., Inc., Kenilworth, New Jersey, USA

*Open Forum Infectious Diseases*, Volume 7, Issue 7, July 2020, ofaa248, https://doi.org/10.1093/ofid/ofaa248

In “Renal Impairment, *C. difficile* Recurrence, and the Differential Effect of Bezlotoxumab: A Post Hoc Analysis of Pooled Data From 2 Randomized Clinical Trials”, *Open Forum Infectious Diseases*, Volume 7, Issue 7, July 2020, ofaa248, few errors occurred in Figure 1 and has been corrected.

**Figure 1.**
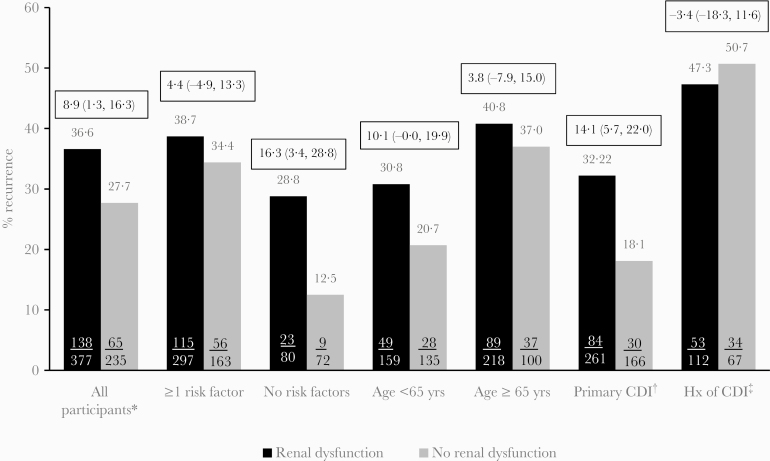
Proportion of placebo-treated participants with rCDI in all participants subset by renal impairment status and in subgroups of participants with prespecified risk factors (Clinical Cure Population). Boxes above bars are differences and 95% confidence intervals. *Clinical Cure Population; †No history of CDI within previous 6 months; ‡History of CDI within previous 6 months; risk factors for rCDI: ≥65 years of age, immunocompromised, history of CDI in previous 6 months, severe CDI (Zar score ≥2), 027 strain.

